# Risk Factors for Colonization With Extended-Spectrum Cephalosporin-Resistant and Carbapenem-Resistant Enterobacterales Among Hospitalized Patients in Kenya: An Antibiotic Resistance in Communities and Hospitals (ARCH) Study

**DOI:** 10.1093/cid/ciad258

**Published:** 2023-07-05

**Authors:** Sylvia Omulo, Teresa Ita, Robert Mugoh, Charchil Ayodo, Ulzii Luvsansharav, Susan Bollinger, Ashley Styczynski, Brooke M Ramay, Mark A Caudell, Guy H Palmer, Samuel Kariuki, Douglas R Call, Rachel M Smith

**Affiliations:** Paul G. Allen School for Global Health, Washington State University, Pullman, Washington, USA; Washington State University Global Health–Kenya, Nairobi, Kenya; University of Nairobi Institute of Tropical and Infectious Diseases, Nairobi, Kenya; Washington State University Global Health–Kenya, Nairobi, Kenya; Washington State University Global Health–Kenya, Nairobi, Kenya; Washington State University Global Health–Kenya, Nairobi, Kenya; Division of Healthcare Quality Promotion, US Centers for Disease Control and Prevention, Atlanta, Georgia, USA; Division of Healthcare Quality Promotion, US Centers for Disease Control and Prevention, Atlanta, Georgia, USA; Division of Healthcare Quality Promotion, US Centers for Disease Control and Prevention, Atlanta, Georgia, USA; Paul G. Allen School for Global Health, Washington State University, Pullman, Washington, USA; Center for Health Studies, Universidad del Valle de Guatemala, Guatemala City, Guatemala; Paul G. Allen School for Global Health, Washington State University, Pullman, Washington, USA; Paul G. Allen School for Global Health, Washington State University, Pullman, Washington, USA; Washington State University Global Health–Kenya, Nairobi, Kenya; University of Nairobi Institute of Tropical and Infectious Diseases, Nairobi, Kenya; Kenya Medical Research Institute, Nairobi, Kenya; Paul G. Allen School for Global Health, Washington State University, Pullman, Washington, USA; Division of Healthcare Quality Promotion, US Centers for Disease Control and Prevention, Atlanta, Georgia, USA

**Keywords:** antimicrobial resistance, colonization, hospitals, Kenya

## Abstract

**Background:**

The spread of extended-spectrum cephalosporin-resistant Enterobacterales (ESCrE) and carbapenem-resistant Enterobacterales (CRE) represents a significant global public health threat. We identified putative risk factors for ESCrE and CRE colonization among patients in 1 urban and 3 rural hospitals in Kenya.

**Methods:**

During a January 2019 and March 2020 cross-sectional study, stool samples were collected from randomized inpatients and tested for ESCrE and CRE. The Vitek2 instrument was used for isolate confirmation and antibiotic susceptibility testing, and least absolute shrinkage and selection operator (LASSO) regression models were used to identify colonization risk factors while varying antibiotic use measures.

**Results:**

Most (76%) of the 840 enrolled participants received ≥1 antibiotic in the 14 days preceding their enrollment, primarily ceftriaxone (46%), metronidazole (28%), or benzylpenicillin-gentamycin (23%). For LASSO models that included ceftriaxone administration, ESCrE colonization odds were higher among patients hospitalized for ≥3 days (odds ratio, 2.32 [95% confidence interval, 1.6–3.37]; *P* < .001), intubated patients (1.73 [1.03–2.91]; *P* = .009), and persons living with human immunodeficiency virus (1.70 [1.03–2.8]; *P* = .029). CRE colonization odds were higher among patients receiving ceftriaxone (odds ratio, 2.23 [95% confidence interval, 1.14–4.38]; *P* = .025) and for every additional day of antibiotic use (1.08 [1.03–1.13]; *P* = .002).

**Conclusions:**

While CRE colonization was strongly associated with ceftriaxone use and duration of antibiotic use, the odds of ESCrE colonization increased with exposure to the hospital setting and invasive medical devices, which may reflect nosocomial transmission. These data suggest several areas where hospitals can intervene to prevent colonization among hospitalized patients, both through robust infection prevention and control practices and antibiotic stewardship programs.

## Teaser

The healthcare risk factors for colonization with extended-spectrum cephalosporin-resistant Enterobacterales and carbapenem-resistant Enterobacterales in Kenya highlight the importance of controlling antimicrobial resistance in hospitals, which may be best accomplished through antibiotic stewardship and hospital infection prevention and control interventions.

In 2019, an estimated 929 000 global deaths were caused by 6 antibiotic-resistant bacteria and another 3.57 million deaths were attributable to antimicrobial-resistant bacteria [1], with the 3 most common infectious agents being *Escherichia coli*, *Staphylococcus aureus*, and *Klebsiella pneumoniae*. The spread and impact of antibiotic-resistant bacteria in parts of the world worsened during the coronavirus disease 2019 pandemic [2, 3]. It was estimated that during the pandemic, 29 400 people died of antimicrobial-resistant infections in the United States, 40% of whom acquired their infection while in the hospital [3]. These deaths were likely attributable, in part, to longer hospital stays and potentially exacerbated by increased antibiotic use and challenges and limitations imposed on infection prevention and control activities and hospital resources during the pandemic.

Extended-spectrum cephalosporin-resistant Enterobacterales (ESCrE) and carbapenem-resistant Enterobacterales (CRE) represent a clinically important group of antibiotic-resistant bacteria given the magnitude of β-lactam use worldwide and few treatment alternatives, particularly in resource-limited settings [4]. Among inpatients receiving antibiotics in 3 Kenyan referral hospitals, 15%–31% of all prescriptions were ceftriaxone [5]. Carbapenems are infrequently used in Kenyan hospitals [5, 6]—accounting for <10% of antibiotic prescriptions in public referral hospitals [5]—and may not always be available or affordable. Newer combination antibiotics used in high-income countries to treat CRE (eg, ceftazidime-avibactam) are not currently widely available in Kenya, leaving few treatment options for infections due to these organisms.

Although routine antibiotic resistance surveillance systems rely largely on microbiology data from clinically suspected infections, ESCrE and CRE prevalence estimates among bacteria recovered from colonization specimens can provide insights into the prevailing burden of antibiotic resistance and emerging mechanisms of resistance, sometimes before they are recognized as pathogens of clinical concern [7]. Furthermore, because colonization with ESCrE and CRE commonly precedes infection in many patients [8–10], evaluation of risk factors for colonization may identify drivers for transmission as colonized patients may represent a reservoir for transmission in hospitals and communities.

These data may in turn represent actionable areas to inform hospital prevention and control activities with the goal of reducing transmission of antibiotic-resistant organisms, as envisioned in Kenya's National Action Plan on prevention and containment of antimicrobial resistance [11]. Building on a recent Antibiotic Resistance in Communities and Hospitals (ARCH) study [12] in Kenya, which reported a higher prevalence of ESCrE and CRE colonization in hospitals (66% and 11%, respectively) than in associated communities (49% and 1%, respectively) [13], we evaluated potential risk factors for colonization with ESCrE and CRE among hospitalized patients in urban and rural settings in Kenya.

## METHODS

### Study Design and Sample Analysis

This cross-sectional study, which happened between January 2019 and March 2020, was part of the ARCH studies conducted across 6 countries to evaluate the population-based prevalence of colonization with clinically significant antimicrobial-resistant organisms. No deviations were made from the main ARCH protocol, and 3 target microorganisms were investigated. Prevalence data on the target microorganisms [13], including descriptions of the population demographics, antibiotic use measures, and microbiological procedures used, have been published elsewhere.

### Study Participants

One urban (Mbagathi County Hospital in Nairobi County) and 3 rural hospitals (Siaya County Referral Hospital, Bondo Subcounty Hospital, and St Elizabeth Hospital in Siaya County) were included in this study. Patients, regardless of age, were selected by simple random sampling from a serialized list of inpatient beds to attain a sample size of 509 rural and 509 urban participants. Patient selection in rural hospitals was proportional to the number of inpatient beds at each hospital [12]. Medical records of eligible participants were screened to exclude patients with severe neutropenia (absolute neutrophil count <500/μL) or active gastrointestinal bleeding.

Informed consent was obtained from adult patients (aged ≥18 years), and adult guardians of unconscious or sedated patients or children <18 years old. Informed assent was also obtained from conscious children aged 7–17 years. Data on potential risk factors for colonization, including duration of hospitalization, admission ward, medical history, invasive procedures, insertion of medical devices, and antibiotic exposure during hospitalization, were abstracted from patient medical records. A stool or rectal swab sample was collected from enrolled individuals per the study protocol, as described elsewhere [13]. Samples were transported to the laboratory, where they were accessioned and processed on the same day.

### Data Collection and Processing

Stool and rectal samples were cultured using commercially prepared HardyCHROM extended-spectrum ß-lactamase agar plates and HardyCHROM CRE agar plates. After incubation (18–24 hours at 37°C), up to 3 morphologically distinct colonies were selected from each extended-spectrum ß-lactamase and CRE plate. All isolates were cultured on tryptic soy agar, archived as glycerol stocks, and identified using a Vitek2 instrument with gram-negative (GN) identification cards. Interpretation of minimum inhibitory concentration values from antibiotic susceptibility testing (AST)–GN71 cards was based on the Clinical and Laboratory Standards Institute guidelines, 2020 [14].

Data from the VITEK2 assays were used to classify individuals as ESCrE positive if their stool samples had ≥1 bacterial isolate classified as nonsusceptible (intermediate or resistant) to ceftriaxone but susceptible to the 3 carbapenems tested (ertapenem, imipenem, and meropenem), and CRE positive if ≥ 1 isolate was classified as nonsusceptible to ≥1 carbapenem.

### Statistical Analysis

Least absolute shrinkage and selection operator (LASSO) logistic regression models [15] were used to test associations between predictor variables and ESCrE or CRE colonization (0 indicates negative; 1, positive) while controlling for factors that could affect both outcomes and risk factors. Predictor variables were treated as independent and were included based on prior association with antimicrobial resistance (these variables were retained for both the ESCrE and CRE models) [16–18]. Antibiotic use, being among the most cited risk factors for antimicrobial-resistant infections in hospital settings, was examined as 3 different measures, including (1) antibiotics taken 2 weeks before enrollment, (2) the total number of antibiotics administered between patient admission and enrollment dates, and (3) whether ceftriaxone was among the antibiotics administered to a patient. These measures were used to construct 3 models for each outcome of interest, colonization with ESCrE or CRE. Odds ratio ranges were then determined for each outcome of interest, representing the range of point estimates across the 3 models.

Similar independent variables, including duration of antibiotic therapy, types of antibiotics administered, comorbid conditions, use of invasive medical devices, history of invasive procedures, patient residence (private residence or healthcare facility), and duration of hospitalization were retained in each of the 3 models. The duration of the hospital stay was dichotomized from a continuous variable (median, 4 days; interquartile range, 2–9 days) into a variable indicating whether patient had been admitted for ≥3 days. Additional variable selection information is provided in the Supplementary Materials. The hospital-specific admission ward was considered a cluster variable because intraclass correlation coefficients indicated that hospital ward accounted for 12% and 8% of the variance in the detection of ESCrE and CRE, respectively. Risk factors that were not specified as independent variables, such as age and history of diabetes, were included as control variables in the models as summarized in Supplementary Table 1.

LASSO regression analysis was used to estimate the effects of putative risk factors on ESCrE and CRE colonization. LASSO regression performs variable selection using a penalty parameter (λ) to “shrink” coefficients that are less relevant to the outcomes (ie, indicated by small coefficients) or that exhibit collinearity to zero. This reduces bias issues associated with model overfitting. Models were run using the *xplogit* command (Stata software, version 17, StataCorp, TX), which applied the cross-fit partialing-out method (or double machine learning) to estimate the effects of predictor variables and to select from potential control variables to be retained in the model. The *xplogit* process runs the LASSO estimation across a series of resampled data sets, 10 in our case, using estimates from previous runs to improve subsequent estimation.

Covariate selection was performed by applying a penalty parameter (λ) to the coefficients of the covariates. To tune λ, a “plugin” estimator was used to determine a value of λ that dominates the noise in the estimating equations to ensure that selected variables had a high probability of belonging to the model [19]. The *vce* option was used to correct standard errors to account for intragroup correlations. Control variables were included to limit the potential confounding effects of variables on both outcomes and predictors to be weighted in the final estimates [20]. Data were cleaned and analyzed using Stata software, version 17 [21].

### Ethical Considerations

This study was approved by the Kenyatta National Hospital/University of Nairobi Ethical Review Committee (no. P164/03/2018) with reliance agreements from the Washington State University Institutional Review Board (no. 16742-001), the Kenya Medical Research Institute, and the Centers for Disease Control and Prevention (no. 7111). This project was licensed by the Kenya National Commission for Science, Technology & Innovation (NACOSTI-P-21-12461).

## RESULTS

A total of 852 participants enrolled at the hospital sites provided a stool sample [13]. Twelve had incomplete participant records and were excluded from the LASSO regression models (Table 1). The remaining participants had been hospitalized for a median of 4 days at the time of enrollment (interquartile range, 2–9 days). Most urban inpatients (85% [319 of 373]) were residents of Nairobi County, and rural inpatients (90% [420 of 467]) were primarily residents of Siaya County. Overall, 56% of patients were female, and 47% were children aged ≤17 years. Forty-four percent of enrolled patients (366 of 840) were from general wards, 43% from pediatric wards, 9% from maternity wards, and the remaining 4% from other wards. Stool samples for 66% (554 of 840) and 11% (94 of 840) of patients yielded an ESCrE and/or CRE isolate, respectively, and 76% of patients (643 of 840) had received ≥1 antibiotic in the 14 days preceding their enrollment. Commonly administered antibiotics included ceftriaxone (46%), metronidazole (28%), and benzylpenicillin-gentamycin (23%). Carbapenem use was not reported in any hospital.

**Table 1.** Summary Statistics for Variables Used in the LASSO Logistic Regression Models for 840 Participants^a^

Variable	Patients, No. (%)^b^
ESCrE positive	
No	286 (34.1)
Yes	554 (66.0)
CRE positive	
No	746 (88.8)
Yes	94 (11.2)
Antibiotics ≤14 d before enrollment	
No	197 (23.5)
Yes	643 (76.6)
Ceftriaxone ≤14 d before enrollment	
No	449 (53.5)
Yes	391 (46.6)
HIV positive	
No	718 (85.5)
Yes	122 (14.5)
Urinary catheter inserted during hospitalization	
No	705 (83.9)
Yes	135 (16.1)
Any intubation	
No	709 (84.4)
Yes	131 (15.6)
Hospitalized for ≥3 inpatient d	
No	255 (30.4)
Yes	585 (69.6)
Transfer from private residence to hospital	
No	142 (16.9)
Yes	698 (83.1)
	median (IQR) [range]
No. of antibiotics administered ≤14 d before enrollment	1 (1–2) [0–4]
Total duration of antibiotic treatment in hospital, d	3 (1–7) [0–38]

Abbreviations: CRE, carbapenem-resistant Enterobacterales; ESCrE, extended-spectrum cephalosporin-resistant Enterobacterales; HIV, human immunodeficiency virus; IQR, interquartile range; LASSO, least absolute shrinkage and selection operator.

Potential control variable information is available in the Supplementary Materials (Supplementary Table 1).

Data represent no. (%) of patients unless otherwise specified.

### Association of ESCrE Colonization With Exposures in the Healthcare Setting

Patients hospitalized for ≥3 days had a higher probability of being ESCrE positive (odds ratio range, 2.31–2.32) across all models; patients intubated during the current hospitalization had 1.64–1.73 times higher odds of being ESCrE positive across all models (Table 2). Patients living with human immunodeficiency virus (HIV) had 1.65–1.70 times higher odds of being ESCrE positive in 2 models (Table 2). The odds of ESCrE colonization were not significantly impacted by exposure to urinary catheters or origin of the patient (ie, private residence or other healthcare facility; Table 2). One measure of antibiotic use—inpatient antibiotic administration within 2 weeks of enrollment—was associated with a 1.56 (95% confidence interval, 1.01-2.40) times higher likelihood of ESCrE colonization (Table 2).

**Table 2.** Risk Factors for Extended-Spectrum Cephalosporin-Resistant Enterobacterales Colonization Based on 840 Participant Records

Risk Factor	Odds Ratio (Robust 95% CI)^a^		
	Model 1	Model 2	Model 3
Antibiotics ≤14 d before enrollment	1.56^b^ (1.01–2.40)	…	…
Total no. of antibiotics in hospital before enrollment	…	1.28 (0.99–1.66)	
Ceftriaxone ≤14 d before enrollment (0 = no; 1 = yes)	…	…	1.21 (0.85–1.71)
Days of antibiotic treatment in hospital before enrollment	1.02 (0.97–1.06)	1.01 (0.97–1.05)	1.03 (0.99–1.07)
HIV positive (0 = no; 1 = yes)	1.62 (0.94–2.80)	1.65^b^ (1.02–2.68)	1.70^b^ (1.03–2.80)
Urinary catheter (0 = no; 1 = yes)	1.20 (0.72–2.01)	1.19 (0.73–1.97)	1.22 (0.72–2.06)
Intubation (0 = no; 1 = yes)	1.66^b^ (1.06–2.59)	1.64^b^ (1.03–2.62)	1.73^b^ (1.03–2.911)
Hospitalized for ≥3 inpatient d (0 = no; 1 = yes)	2.31^c^ (1.62–3.28)	2.32^c^ (1.68–3.21)	2.32^c^ (1.60–3.37)
Transfer from private residence to hospital^d^	1.17 (0.76–1.80)	1.13 (0.75–1.71)	1.16 (0.74–1.81)

Abbreviations: CI, confidence interval; HIV, human immunodeficiency virus.

Models 1–3 incorporated 3 different measures of antibiotic exposure. Information on the control variables retained for least absolute shrinkage and selection operator (LASSO) estimation is provided in the Supplementary Materials (Supplementary Tables 2–25).

*P* < .05.

*P* < .01.

See Table 1.

### Association of CRE Colonization With Antibiotic Use

The only factor significantly associated with the odds of CRE colonization across all models was the total number of days antibiotics were administered to a patient. Each additional day of antibiotic administration increased the chances of CRE colonization by 1.07–1.08 times (Table 3). For patients receiving ceftriaxone, the odds of CRE colonization were 2.23 (95% confidence interval, 1.14–4.38) times greater than in those who did not receive ceftriaxone. All other factors—HIV status, insertion of a urinary catheter, intubation, hospital stays ≥3 day, and patient residence—were not significantly associated with CRE carriage (Table 3).

**Table 3.** Risk Factors for and Carbapenem-Resistant Enterobacterales Colonization Based on 840 Participant Records

Risk Factor	Odds Ratio (Robust 95% CI)^a^		
	Model 1	Model 2	Model 3
Antibiotics ≤14 d before enrollment	1.55 (0.63–3.84)	…	…
Total no. of antibiotics administered ≤14 d before enrollment	…	1.23 (0.79–1.92)	…
Ceftriaxone ≤14 d before enrollment (0 = no; 1 = yes)	…	…	2.23^b^ (1.14–4.38)
Total days of antibiotic treatment in hospital before enrollment	1.08^c^ (1.04–1.13)	1.07^b^ (1.00–1.14)	1.08^d^ (1.03–1.13)
HIV positive (0 = no; 1 = yes)	0.63 (0.24–1.68)	0.56 (0.17–1.98)	0.54 (0.21–1.39)
Urinary catheter (0 = no; 1 = yes)	0.74 (0.40–1.36)	0.70 (0.34–1.46)	0.70 (0.36–1.39)
Intubation (0 = no; 1 = yes)	1.00 (0.53–1.90)	0.98 (0.46–2.10)	1.03 (0.51–2.08)
Hospitalized for ≥3 inpatient d (0 = no; 1 = yes)	1.53 (0.72–3.28)	1.56 (0.66–3.67)	1.41 (0.61–3.24)
Transfer from elsewhere^e^	0.99 (0.52–1.87)	0.97 (0.45–2.06)	0.91 (0.46–1.80)

Abbreviations: CI, confidence interval; HIV, human immunodeficiency virus.

Models 1–3 incorporated 3 different measures of antibiotic exposure. Information on the control variables retained for least absolute shrinkage and selection operator (LASSO) estimation are provided in the Supplementary Materials (Supplementary Tables 25–49).

*P* < .05.

*P* < .001.

*P* < .01,

See Table 1.

## DISCUSSION

This study sought to identify the drivers of colonization with ESCrE and CRE in the Kenyan hospital context, with the aim of identifying potential areas of intervention to reduce colonization acquisition which can lead to infection and transmission. We observed differences in the importance of non–antibiotic-related risk factors between ESCrE- and CRE-colonized patients. The main risk factors for ESCrE colonization in our study population included primarily non–antibiotic-related risk factors (Table 2), although one of the models (model 1) identified recent antibiotic administration as an independent risk factor; these data are consistent with risk factors identified in studies of ESCrE infections [10, 22, 23]. For CRE, the risk of colonization was more closely associated with antibiotic use measures across all models.

The higher risk of ESCrE colonization among patients with ≥3 days of hospitalization highlights the importance of the healthcare setting as a risk for colonization. This may represent nosocomial transmission facilitated via patient-to-patient contact, healthcare worker-to-patient contact, or fomites [24], especially where infection prevention and control practices are poor. In our study, intubation was a significant risk factor for ESCrE colonization across all models. Intubation may be a marker for sick patients who may be more susceptible to colonization due to impaired immune response or dysbiosis. Endotracheal tubes are among the most common invasive devices among hospitalized patients in public hospitals in Kenya [5]. Although urinary catheters have also been implicated in the acquisition of multidrug-resistant bacteria in urinary tract infections [22], they were not a significant risk factor for ESCrE colonization in this study.

Most literature on HIV and colonization with multidrug-resistant nontuberculous bacteria focuses on high rates of methicillin-resistant *S. aureus* colonization and infection among persons living with HIV [25, 26]. Even though the World Health Organization estimates that 38.4 million (33.9–43.8 million) people were living with HIV in 2021 [27], data on colonization with ESCrE and CRE in persons living with HIV are limited. Our study identified HIV positivity as an independent risk factor for ESCrE colonization. HIV-positive individuals may be at risk of ESCrE colonization owing to several factors. Those with poorly controlled antiretroviral therapy (ART) may be immunocompromised, leading to increased contact with healthcare (including hospitalizations) and opportunities for colonization. Nevertheless, even among persons well controlled by ART, studies demonstrate the persistence of dysbiosis and inflammation in the gut microbiome, which may also predispose to acquisition of resistant organisms [28, 29]. Furthermore, the use of prophylactic antibiotics such as sulfamethoxazole-trimethoprim among HIV-infected persons may coselect for antibiotic-resistant Enterobacterales strains. Given the global number of persons living with HIV and the increasing spread of antimicrobial resistance, a better understanding of the way in which HIV predisposes to colonization with resistant organisms is important in order to mitigate morbidity and mortality rates in this high-risk group. For example, azidothymidine has been shown to have antibiotic effects [30]

The association between CRE colonization and antibiotic use across all models along with the absence of other associations suggest that CRE colonization may be driven primarily by antibiotic-related sequelae, such as selection pressure for resistant organisms or changes to the microbiome leading to dysbiosis and increased susceptibility to colonization. In our study, the use of ceftriaxone, which can select for CRE [31], posed the greatest odds for colonization (model 3). Ceftriaxone is the most prescribed antibiotic among inpatients in many public healthcare facilities in Kenya [5, 32, 33], and was administered to almost half (46%) of the patients in our study. Understanding the appropriate level of use of ceftriaxone and other broad-spectrum antibiotics is critical to mitigate the development and spread of resistance. Robust antibiotic stewardship programs can help ensure appropriate use of antibiotics in these healthcare facilities.

We acknowledge several limitations of this study. Its cross-sectional nature did not allow us to determine whether ESCrE- or CRE-colonized patients were likely to require longer hospitalization or more antibiotic administration, respectively, or whether these factors contributed to their colonization during hospitalization (ie, the actual temporal relationship between outcomes and risk factors). Disentangling these relationships will require longitudinal study designs that include other important factors not collected in the current study, such as previous hospitalizations. In addition, despite the inclusion of different hospitals and different wards, we did not collect data on ward-specific or hospital-specific risk factors, which are critical to understanding prevention activities that are broader than those that focus on the patient alone. We did not collect data on HIV control or measures of immunosuppression, so we were unable to evaluate whether the association of ESCrE colonization and HIV positivity was influenced by ART or metrics of HIV severity. Finally, as in most epidemiological studies, the risk factors identified in our study could be surrogates for other causative factors that were not otherwise accounted for.

In conclusion, the risk factors for colonization identified in this study—limiting the use of invasive devices, judicious use of antibiotics, and reducing the transmission risk—are similar to those associated with nosocomial infections. This emphasizes not only the importance of infection prevention and control activities but also the utility of colonization data—rather than data on nosocomial infections—in evaluating the effectiveness of hospital interventions. Further research, particularly longitudinal, is needed to parse out the association between HIV and colonization with ESCrE, with attention to measures of disease severity and control (eg, CD4 cell count and viral load). Intervening to prevent colonization with ESCrE and CRE will not only benefit the individual patient, by reducing the risk of infection, but will also help reduce the overall colonization pressure within a facility, leading to decreases in nosocomial transmission more broadly and reduced healthcare costs.

## Supplementary Data

Supplementary materials are available at *Clinical Infectious Diseases* online. Consisting of data provided by the authors to benefit the reader, the posted materials are not copyedited and are the sole responsibility of the authors, so questions or comments should be addressed to the corresponding author.

## Supplementary Material

ciad258_Supplementary_Data

## Notes

***Acknowledgments.*** Assistance and support were provided by hospital administrators, county administration (Nairobi and Siaya), the Ministry of Health–Kenya, the Diagnostics and Laboratory Systems Program (Kisumu), and the University of Nairobi Institute of Tropical Infectious Diseases laboratory (Nairobi). J. Horton provided additional laboratory support.

***Financial support.*** This work was supported by the Centers for Disease Control and Prevention, United States (grant U01GH002143 to D.R.C.).

***Supplement sponsorship.*** This article appears as part of the supplement “The Evolving Challenges of Antibiotic Resistance in Low- and Middle-Income Countries: Priorities and Solutions,” sponsored by the U.S. Centers for Disease Control and Prevention, and Health Security Partners.
