# Supplementary material for: Risk Factors for Colonization With Extended-Spectrum Cephalosporin-Resistant and Carbapenem-Resistant Enterobacterales Among Hospitalized Patients in Kenya: An Antibiotic Resistance in Communities and Hospitals (ARCH) Study
Source: Clin Infect Dis. 2023 Jul 5;77(Suppl 1):S97–S103. doi: 10.1093/cid/ciad258 (PMC10321688; doi:10.1093/cid/ciad258)
Supplement: ciad258_Supplementary_Data [file ciad258_supplementary_data.docx]

**Supplementary file**

**Table S1**. **Control variables used in the LASSO models**

| **Variable Name** | **Variable Label** | **Label** | **Code** |
| --- | --- | --- | --- |
| **CATEGORICAL** | | | |
| ippartsex | Participant sex | Male | 1 |
|  |  | Female | 2 |
| rural_urban | Hospital in rural or urban area | Rural | 0 |
|  |  | Urban | 1 |
| Amoxicillin | Antibiotic administered to patient | No | 0 |
|  |  | Yes | 1 |
| Benzylpenicillin | Antibiotic administered to patient | No | 0 |
|  |  | Yes | 1 |
| Ceftazidime | Antibiotic administered to patient | No | 0 |
|  |  | Yes | 1 |
| Erythromycin | Antibiotic administered to patient | No | 0 |
|  |  | Yes | 1 |
| Flucloxacillin | Antibiotic administered to patient | No | 0 |
|  |  | Yes | 1 |
| Gentamicin | Antibiotic administered to patient | No | 0 |
|  |  | Yes | 1 |
| Metronidazole_oral_rectal | Antibiotic administered to patient | No | 0 |
|  |  | Yes | 1 |
| Metronidazole_parenteral | Antibiotic administered to patient | No | 0 |
|  |  | Yes | 1 |
| diabetes | Patient had diabetes | No | 0 |
|  |  | Yes | 1 |
| liver_disease | Patient had liver disease | No | 0 |
|  |  | Yes | 1 |
| malignancy | Patient had malignancy | No | 0 |
|  |  | Yes | 1 |
| kidney_disease | Patient had kidney disease | No | 0 |
|  |  | Yes | 1 |
| neurologic_disease | Patient had neurologic disease | No | 0 |
|  |  | Yes | 1 |
| steroid_use | Patient used steroids | No | 0 |
|  |  | Yes | 1 |
| cardiovascular_disease | Patient had cardiovasculardisease | No | 0 |
|  |  | Yes | 1 |
| respiratory_disease | Patient had respiratory disease | No | 0 |
|  |  | Yes | 1 |
| tb | Patient had tuberculosis | No | 0 |
|  |  | Yes | 1 |
| Severe_neutropenia | Patient had severe neutropenia | No | 0 |
|  |  | Yes | 1 |
| none | No Chronic diseases | No | 0 |
|  |  | Yes | 1 |
| Other | Other Chronic Diseases | No | 0 |
|  |  | Yes | 1 |
| **NON-CATEGORICAL** | | | |
| ipartage | Participant’s age | Number |  |
| invasivetheatrenum | Number of invasive procedures | Number |  |
| invasiveoutnum | Number of invasive procedures not performed in operating room hospitalization | Number |  |

**Note:** Immunosuppression, including history of corticosteroid use, use of traditional chemotherapy and HIV were explored as risk factors. Of the 840 patients considered in the analysis, one was using corticosteroids, 17 had cancer (chemotherapy administration at time of enrollment was not established) and 122 were HIV-positive. To avoid spurious conclusions about general immunosuppression, a minor proportion of the population was excluded leaving only the HIV-positive population.

**II. Control variables retained in LASSO estimation for ESCrE Models.** K-fold cross-validation randomly splits the data into 10 parts, with 9 used for training and one for testing. Categorical variables are presented first by variable name and then category(ies) in the subsequent row. For example, in Table S27, “Benzylpenicillin” is the variable name and “No” indicates respondents who were not given benzylpenicillin.

**Table S2. Control variables retained in ESCrE Model 1 across 10 folds.** ESCrE outcome variable.

|  | 1 | 2 | 3 | 4 | 5 | 6 | 7 | 8 | 9 | 10 |
| --- | --- | --- | --- | --- | --- | --- | --- | --- | --- | --- |
| _cons | x | x | x | x | x | x | x | x | x | x |

**Table S3. Control variables retained in ESCrE Model 1 across 10 folds.** Antibiotic use in last 2 weeks.

|  | 1 | 2 | 3 | 4 | 5 | 6 | 7 | 8 | 9 | 10 |
| --- | --- | --- | --- | --- | --- | --- | --- | --- | --- | --- |
| Benzylpenicillin |  |  |  |  |  |  |  |  |  |  |
| No | x | x | x | x | x | x | x | x | x | x |
| Gentamicin |  |  |  |  |  |  |  |  |  |  |
| No | x |  |  | x |  | x | x |  |  | x |
| Yes |  | x | x |  | x |  |  | x | x |  |
| Metronidazole_parenteral |  |  |  |  |  |  |  |  |  |  |
| No | x | x | x | x | x | x | x | x | x | x |
| Steroid_use |  |  |  |  |  |  |  |  |  |  |
| No |  |  | x |  |  |  |  | x |  |  |
| _cons | x | x | x | x | x | x | x | x | x | x |

**Table S4. Control variables retained in ESCrE Model 1 across 10 folds.** Number of days antibiotics administered.

|  | 1 | 2 | 3 | 4 | 5 | 6 | 7 | 8 | 9 | 10 |
| --- | --- | --- | --- | --- | --- | --- | --- | --- | --- | --- |
| Severe neutropenia |  |  |  |  |  |  |  |  |  |  |
| No | x | x | x |  |  |  |  | x |  | x |
| Not recorded | x | x |  |  | x | x |  |  | x | x |
| Flucloxacillin |  |  |  |  |  |  |  |  |  |  |
| No | x | x | x | x | x | x | x | x | x | x |
| Metronidazole_parenteral |  |  |  |  |  |  |  |  |  |  |
| No | x | x | x | x | x | x | x | x | x | x |
| _cons | x | x | x | x | x | x | x | x | x | x |

**Table S5. Control variables retained in ESCrE Model 1 across 10 folds.** HIV.

|  | 1 | 2 | 3 | 4 | 5 | 6 | 7 | 8 | 9 | 10 |
| --- | --- | --- | --- | --- | --- | --- | --- | --- | --- | --- |
| TB |  |  |  |  |  |  |  |  |  |  |
| No | x | x | x | x | x | x | x | x | x | x |
| No Chronic Disease |  |  |  |  |  |  |  |  |  |  |
| No | x | x | x | x | x | x | x | x | x | x |
| _cons | x | x | x | x | x | x | x | x | x | x |

**Table S6. Control variables retained in ESCrE Model 1 across 10 folds.** Urinary catheter.

|  | 1 | 2 | 3 | 4 | 5 | 6 | 7 | 8 | 9 | 10 |
| --- | --- | --- | --- | --- | --- | --- | --- | --- | --- | --- |
| ippartagegp3 | x |  | x | x | x | x | x | x |  | x |
| Severe neutropenia |  |  |  |  |  |  |  |  |  |  |
| No | x | x | x | x |  | x |  | x | x | x |
| Metronidazole_parenteral |  |  |  |  |  |  |  |  |  |  |
| No |  |  |  | x |  |  |  |  |  |  |
| Unknown antibiotics |  |  |  |  |  |  |  |  |  |  |
| No |  |  |  |  |  |  |  | x | x |  |
| _cons | x | x | x | x | x | x | x | x | x | x |

**Table S7. Control variables retained in ESCrE Model 1 across 10 folds.** Intubated.

|  | 1 | 2 | 3 | 4 | 5 | 6 | 7 | 8 | 9 | 10 |
| --- | --- | --- | --- | --- | --- | --- | --- | --- | --- | --- |
| Ceftazidime |  |  |  |  |  |  |  |  |  |  |
| No | x | x | x | x |  | x | x | x | x | x |
| _cons | x | x | x | x | x | x | x | x | x | x |

**Table S8. Control variables retained in ESCrE Model 1 across 10 folds.** Hospital stay for ≥3 days.

|  | 1 | 2 | 3 | 4 | 5 | 6 | 7 | 8 | 9 | 10 |
| --- | --- | --- | --- | --- | --- | --- | --- | --- | --- | --- |
| Erythromycin |  |  |  |  |  |  |  |  |  |  |
| No | x | x |  |  | x | x | x | x | x | x |
| _cons | x | x | x | x | x | x | x | x | x | x |

**Table S9. Control variables retained in ESCrE Model 1 across 10 folds.** Transferred from another facility.

|  | 1 | 2 | 3 | 4 | 5 | 6 | 7 | 8 | 9 | 10 |
| --- | --- | --- | --- | --- | --- | --- | --- | --- | --- | --- |
| ipgastrobleed |  |  |  |  |  |  |  |  |  |  |
| No | x |  | x | x | x | x |  |  | x | x |
| kidney_disease |  |  |  |  |  |  |  |  |  |  |
| No | x |  | x | x | x |  | x |  |  | x |
| Yes |  |  |  |  |  |  |  | x | x |  |
| Severe neutropenia |  |  |  |  |  |  |  |  |  |  |
| No |  |  |  |  | x |  |  |  |  |  |
| _cons | x | x | x | x | x | x | x | x | x | x |

**Table S10. Control variables retained in ESCrE Model 2 across 10 folds.** ESCrE outcome variable.

|  | 1 | 2 | 3 | 4 | 5 | 6 | 7 | 8 | 9 | 10 |
| --- | --- | --- | --- | --- | --- | --- | --- | --- | --- | --- |
| _cons | x | x | x | x | x | x | x | x | x | x |

**Table S11. Control variables retained in ESCrE Model 2 across 10 folds.** Total antibiotics given.

|  | 1 | 2 | 3 | 4 | 5 | 6 | 7 | 8 | 9 | 10 |
| --- | --- | --- | --- | --- | --- | --- | --- | --- | --- | --- |
| Flucloxacillin |  |  |  |  |  |  |  |  |  |  |
| No | x | x | x | x | x | x | x | x | x | x |
| Metronidazole_parenteral |  |  |  |  |  |  |  |  |  |  |
| No | x | x | x | x | x | x | x | x | x | x |
| Benzylpenicillin |  |  |  |  |  |  |  |  |  |  |
| No |  | x |  |  |  |  |  |  |  |  |
| Gentamicin |  |  |  |  |  |  |  |  |  |  |
| Yes |  | x |  |  |  |  |  |  |  |  |
| Erythromycin |  |  |  |  |  |  |  |  |  |  |
| No |  |  | x |  | x |  |  |  |  |  |
| Yes |  |  |  |  | x |  |  |  |  |  |
| _cons | x | x | x | x | x | x | x | x | x | x |

**Table S12. Control variables retained in ESCrE Model 2 across 10 folds.** Number of days antibiotics administered.

|  | 1 | 2 | 3 | 4 | 5 | 6 | 7 | 8 | 9 | 10 |
| --- | --- | --- | --- | --- | --- | --- | --- | --- | --- | --- |
| Severe neutropenia |  |  |  |  |  |  |  |  |  |  |
| No | x | x | x |  |  | x |  |  | x | x |
| Not recorded | x |  | x |  | x |  |  | x |  |  |
| Flucloxacillin |  |  |  |  |  |  |  |  |  |  |
| No | x | x | x | x | x | x | x | x | x | x |
| Yes |  | x |  |  |  |  |  |  |  |  |
| Metronidazole_parenteral |  |  |  |  |  |  |  |  |  |  |
| No | x | x | x | x | x | x | x | x | x | x |
| _cons | x | x | x | x | x | x | x | x | x | x |

**Table S13. Control variables retained in ESCrE Model 2 across 10 folds**. HIV.

|  | 1 | 2 | 3 | 4 | 5 | 6 | 7 | 8 | 9 | 10 |
| --- | --- | --- | --- | --- | --- | --- | --- | --- | --- | --- |
| TB |  |  |  |  |  |  |  |  |  |  |
| No | x | x | x | x | x | x | x | x | x | x |
| No Chronic Disease |  |  |  |  |  |  |  |  |  |  |
| No | x | x | x | x | x | x | x | x | x | x |
| _cons | x | x | x | x | x | x | x | x | x | x |

**Table S14. Control variables retained in ESCrE Model 2 across 10 folds.** Urinary Catheter.

|  | 1 | 2 | 3 | 4 | 5 | 6 | 7 | 8 | 9 | 10 |
| --- | --- | --- | --- | --- | --- | --- | --- | --- | --- | --- |
| ippartagegp3 | x |  | x | x | x | x | x | x |  | x |
| Severe neutropenia |  |  |  |  |  |  |  |  |  |  |
| No | x | x | x | x |  | x |  | x | x | x |
| Metronidazole_parenteral |  |  |  |  |  |  |  |  |  |  |
| No |  |  |  | x |  |  |  |  |  |  |
| Amoxicillin |  |  |  |  |  |  |  |  |  |  |
| Yes |  |  |  |  |  |  | x |  |  |  |
| Unknown antibiotics |  |  |  |  |  |  |  |  |  |  |
| No |  |  |  |  |  |  |  | x | x |  |
| _cons | x | x | x | x | x | x | x | x | x | x |

**Table S15. Control variables retained in ESCrE Model 2 across 10 folds.** Intubated.

|  | 1 | 2 | 3 | 4 | 5 | 6 | 7 | 8 | 9 | 10 |
| --- | --- | --- | --- | --- | --- | --- | --- | --- | --- | --- |
| Ceftazidime |  |  |  |  |  |  |  |  |  |  |
| No | x | x | x | x |  | x | x | x | x | x |
| _cons | x | x | x | x | x | x | x | x | x | x |

**Table S16. Control variables retained in ESCrE Model 2 across 10 folds.** Hospital stay for ≥3 days.

|  | 1 | 2 | 3 | 4 | 5 | 6 | 7 | 8 | 9 | 10 |
| --- | --- | --- | --- | --- | --- | --- | --- | --- | --- | --- |
| Erythromycin |  |  |  |  |  |  |  |  |  |  |
| No | x | x |  |  | x | x | x | x | x | x |
| _cons | x | x | x | x | x | x | x | x | x | x |

**Table S17. Control variables retained in ESCrE Model 2 across 10 folds.** Transferred from another facility.

|  | 1 | 2 | 3 | 4 | 5 | 6 | 7 | 8 | 9 | 10 |
| --- | --- | --- | --- | --- | --- | --- | --- | --- | --- | --- |
| ipgastrobleed |  |  |  |  |  |  |  |  |  |  |
| No | x |  | x | x | x | x |  |  | x | x |
| kidney_disease |  |  |  |  |  |  |  |  |  |  |
| No | x |  | x | x |  |  | x | x |  | x |
| Yes |  | x |  |  | x |  |  |  | x |  |
| Severe neutropenia |  |  |  |  | x |  |  |  |  |  |
| No |  |  |  |  |  |  |  |  |  |  |
| _cons | x | x | x | x | x | x | x | x | x | x |

**Table S18. Control variables retained in ESCrE Model 3 across 10 folds.** ESCrE outcome variable.

|  | 1 | 2 | 3 | 4 | 5 | 6 | 7 | 8 | 9 | 10 |
| --- | --- | --- | --- | --- | --- | --- | --- | --- | --- | --- |
| _cons | x | x | x | x | x | x | x | x | x | x |

**Table S19. Control variables retained in ESCrE Model 3 across 10 folds.** Ceftriaxone use.

|  | 1 | 2 | 3 | 4 | 5 | 6 | 7 | 8 | 9 | 10 |
| --- | --- | --- | --- | --- | --- | --- | --- | --- | --- | --- |
| Metronidazole_parenteral |  |  |  |  |  |  |  |  |  |  |
| No |  | x | x | x |  | x | x | x | x | x |
| Erythromycin |  |  |  |  |  |  |  |  |  |  |
| Yes |  |  | x |  |  |  |  |  |  |  |
| Benzylpenicillin |  |  |  |  |  |  |  |  |  |  |
| No |  |  |  | x |  |  |  |  |  |  |
| Kidney_disease |  |  |  |  |  |  |  |  |  |  |
| No |  |  |  |  |  | x |  |  |  |  |
| Ceftazidime |  |  |  |  |  |  |  |  |  |  |
| No |  |  |  |  |  |  | x |  |  |  |
| _cons | x | x | x | x | x | x | x | x | x | x |

**Table S20. Control variables retained in ESCrE Model 3 across 10 folds.** Number of days antibiotics administered.

|  | 1 | 2 | 3 | 4 | 5 | 6 | 7 | 8 | 9 | 10 |
| --- | --- | --- | --- | --- | --- | --- | --- | --- | --- | --- |
| Severe neutropenia |  |  |  |  |  |  |  |  |  |  |
| No | x | x | x |  | x | x |  | x | x | x |
| Not recorded |  | x |  |  |  |  |  |  |  |  |
| Flucloxacillin |  |  |  |  |  |  |  |  |  |  |
| No | x | x | x | x | x | x | x | x | x | x |
| Metronidazole_parenteral |  |  |  |  |  |  |  |  |  |  |
| No | x | x | x | x | x | x | x | x | x | x |
| _cons | x | x | x | x | x | x | x | x | x | x |

**Table S21. Control variables retained in ESCrE Model 3 across 10 folds**. HIV.

|  | 1 | 2 | 3 | 4 | 5 | 6 | 7 | 8 | 9 | 10 |
| --- | --- | --- | --- | --- | --- | --- | --- | --- | --- | --- |
| TB |  |  |  |  |  |  |  |  |  |  |
| No | x | x | x | x | x | x | x | x | x | x |
| No Chronic Disease |  |  |  |  |  |  |  |  |  |  |
| No | x | x | x | x | x | x | x | x | x | x |
| _cons | x | x | x | x | x | x | x | x | x | x |

**Table S22. Control variables retained in ESCrE Model 3 across 10 folds.** Urinary Catheter.

|  | 1 | 2 | 3 | 4 | 5 | 6 | 7 | 8 | 9 | 10 |
| --- | --- | --- | --- | --- | --- | --- | --- | --- | --- | --- |
| ippartagegp3 | x |  | x | x | x | x | x | x |  | x |
| Severe neutropenia |  |  |  |  |  |  |  |  |  |  |
| No | x | x | x | x |  | x |  | x | x | x |
| Metronidazole_parenteral |  |  |  |  |  |  |  |  |  |  |
| No |  |  |  | x |  |  |  |  |  |  |
| Unknown antibiotic |  |  |  |  |  |  |  |  |  |  |
| No |  |  |  |  |  |  |  | x | x |  |
| _cons | x | x | x | x | x | x | x | x | x | x |

**Table S23. Control variables retained in ESCrE Model 3 across 10 folds.** Intubated.

|  | 1 | 2 | 3 | 4 | 5 | 6 | 7 | 8 | 9 | 10 |
| --- | --- | --- | --- | --- | --- | --- | --- | --- | --- | --- |
| Ceftazidime |  |  |  |  |  |  |  |  |  |  |
| No | x | x | x | x |  | x | x |  | x | x |
| _cons | x | x | x | x | x | x | x | x | x | x |

**Table S24. Control variables retained in ESCrE Model 3 across 10 folds.** Hospital stay for ≥3 days.

|  | 1 | 2 | 3 | 4 | 5 | 6 | 7 | 8 | 9 | 10 |
| --- | --- | --- | --- | --- | --- | --- | --- | --- | --- | --- |
| Erythromycin |  |  |  |  |  |  |  |  |  |  |
| No | x | x |  |  | x | x | x | x | x | x |
| _cons | x | x | x | x | x | x | x | x | x | x |

**Table S25. Control variables retained in ESCrE Model 3 across 10 folds.** Transferred from another facility.

|  | 1 | 2 | 3 | 4 | 5 | 6 | 7 | 8 | 9 | 10 |
| --- | --- | --- | --- | --- | --- | --- | --- | --- | --- | --- |
| Ipgastrobleed |  |  |  |  |  |  |  |  |  |  |
| No | x |  | x | x | x | x |  |  | x |  |
| Kidney_disease |  |  |  |  |  |  |  |  |  |  |
| Yes | x |  | x |  | x |  |  |  | x | x |
| No |  |  |  | x |  |  | x | x |  |  |
| Severe neutropenia |  |  |  |  |  |  |  |  |  |  |
| No |  |  |  |  | x |  |  |  |  |  |
| _cons | x | x | x | x | x | x | x | x | x | x |

**III. Control variables retained in Lasso estimation for ESCrE Models**. K-fold cross validation randomly splits the data into 10 parts, with 9 used for training and one for testing. Categorical variables are presented first by variable name and then category(ies) in the subsequent row. For example, in Table S3, “Benzylpenicillin” is the variable name and “No” indicates respondents who were not given benzylpenicillin.

**Table S26. Control variables retained in CRE Model 1 across 10 folds:** CRE outcome variable.

|  | 1 | 2 | 3 | 4 | 5 | 6 | 7 | 8 | 9 | 10 |
| --- | --- | --- | --- | --- | --- | --- | --- | --- | --- | --- |
| _cons | x | x | x | x | x | x | x | x | x | x |

**Table S27. Control variables retained in CRE Model 1 across 10 folds:** Antibiotic use in last 2 weeks.

|  | 1 | 2 | 3 | 4 | 5 | 6 | 7 | 8 | 9 | 10 |
| --- | --- | --- | --- | --- | --- | --- | --- | --- | --- | --- |
| Benzylpenicillin |  |  |  |  |  |  |  |  |  |  |
| No | x | x | x | x | x | x | x | x | x | x |
| Gentamicin |  |  |  |  |  |  |  |  |  |  |
| No | x | x |  |  | x |  |  |  | x |  |
| Yes |  |  | x | x |  | x | x | x |  | x |
| Metronidazole_parenteral |  |  |  |  |  |  |  |  |  |  |
| No | x | x | x | x | x | x | x | x | x | x |
| Flucloxacillin |  |  |  |  |  |  |  |  |  |  |
| No |  |  |  |  |  |  |  |  | x |  |
| _cons | x | x | x | x | x | x | x | x | x | x |

**Table S28. Control variables retained in CRE Model 1 across 10 folds:** Number of days antibiotics administered

|  | 1 | 2 | 3 | 4 | 5 | 6 | 7 | 8 | 9 | 10 |
| --- | --- | --- | --- | --- | --- | --- | --- | --- | --- | --- |
| Severe neutropenia |  |  |  |  |  |  |  |  |  |  |
| No | x | x | x | x |  | x | x | x |  | x |
| Not recorded |  |  |  |  | x |  |  |  | x |  |
| Steroid_use |  |  |  |  |  |  |  |  |  |  |
| No | x | x |  | x |  | x | x |  |  |  |
| Flucloxacillin |  |  |  |  |  |  |  |  |  |  |
| No | x | x | x | x | x | x | x |  | x | x |
| Yes |  |  |  |  |  |  |  | x |  |  |
| Metronidazole_parenteral |  |  |  |  |  |  |  |  |  |  |
| No | x | x | x | x | x | x | x | x | x | x |
| _cons | x | x | x | x | x | x | x | x | x | x |

**Table S29. Control variables retained in CRE Model 1 across 10 folds:** HIV

|  | 1 | 2 | 3 | 4 | 5 | 6 | 7 | 8 | 9 | 10 |
| --- | --- | --- | --- | --- | --- | --- | --- | --- | --- | --- |
| Severe neutropenia |  |  |  |  |  |  |  |  |  |  |
| No | x | x | x |  |  | x |  | x | x | x |
| TB |  |  |  |  |  |  |  |  |  |  |
| No | x | x | x | x | x | x | x | x | x | x |
| No Chronic Disease |  |  |  |  |  |  |  |  |  |  |
| No | x | x | x | x | x | x | x | x | x | x |
| _cons | x | x | x | x | x | x | x | x | x | x |

**Table S30. Control variables retained in CRE Model 1 across 10 folds:** Urinary Catheter

|  | 1 | 2 | 3 | 4 | 5 | 6 | 7 | 8 | 9 | 10 |
| --- | --- | --- | --- | --- | --- | --- | --- | --- | --- | --- |
|  |  |  |  |  |  |  |  |  |  |  |
| ippartagegp3 | x |  | x | x | x | x | x | x | x | x |
| severe neutropenia |  |  |  |  |  |  |  |  |  |  |
| No | x |  | x | x |  |  |  |  |  | x |
| Metronidazole_parenteral |  |  |  |  |  |  |  |  |  |  |
| No | x |  | x | x |  |  |  |  |  | x |
| Amoxicillin |  |  |  |  |  |  |  |  |  |  |
| No |  |  |  |  |  |  | x |  |  |  |
| _cons | x | x | x | x | x | x | x | x | x | x |

**Table S31. Control variables retained in CRE Model 1 across 10 folds:** Intubated

|  | 1 | 2 | 3 | 4 | 5 | 6 | 7 | 8 | 9 | 10 |
| --- | --- | --- | --- | --- | --- | --- | --- | --- | --- | --- |
| Ceftazidime |  |  |  |  |  |  |  |  |  |  |
| No | x | x | x | x |  | x | x | x | x | x |
| _cons | x | x | x | x | x | x | x | x | x | x |

**Table S32. Control variables retained in CRE Model 1 across 10 folds:** Hospital stay for ≥3 days.

|  | 1 | 2 | 3 | 4 | 5 | 6 | 7 | 8 | 9 | 10 |
| --- | --- | --- | --- | --- | --- | --- | --- | --- | --- | --- |
| Erythromycin |  |  |  |  |  |  |  |  |  |  |
| No | x | x |  | x | x | x | x | x | x | x |
| Ceftazidime |  |  |  |  |  |  |  |  |  |  |
| No |  |  |  |  | x |  |  |  |  |  |
| _cons | x | x | x | x | x | x | x | x | x | x |

**Table S33. Control variables retained in CRE Model 1 across 10 folds.** Transferred from another facility

|  | 1 | 2 | 3 | 4 | 5 | 6 | 7 | 8 | 9 | 10 |
| --- | --- | --- | --- | --- | --- | --- | --- | --- | --- | --- |
| Kidney_disease |  |  |  |  |  |  |  |  |  |  |
| No | x | x | x | x | x |  | x | x | x |  |
| Yes |  |  |  |  |  |  |  |  |  | x |
| Severe neutropenia |  |  |  |  |  |  |  |  |  |  |
| No |  |  |  |  | x |  |  |  |  |  |
| Erythromycin |  |  |  |  |  |  |  |  |  |  |
| Yes |  |  |  |  | x |  |  |  |  |  |
| No |  |  |  |  |  |  |  |  |  | x |
| _cons | x | x | x | x | x | x | x | x | x | x |

**Table S34. Control variables retained in CRE Model 2 across 10 folds.** CRE outcome variable.

|  | 1 | 2 | 3 | 4 | 5 | 6 | 7 | 8 | 9 | 10 |
| --- | --- | --- | --- | --- | --- | --- | --- | --- | --- | --- |
| _cons | x | x | x | x | x | x | x | x | x | x |

**Table S35. Control variables retained in CRE Model 2 across 10 folds.** Total antibiotics given.

|  | 1 | 2 | 3 | 4 | 5 | 6 | 7 | 8 | 9 | 10 |
| --- | --- | --- | --- | --- | --- | --- | --- | --- | --- | --- |
| Flucloxacillin |  |  |  |  |  |  |  |  |  |  |
| No | x | x | x | x | x | x | x | x | x | x |
| Metronidazole_parenteral |  |  |  |  |  |  |  |  |  |  |
| No | x | x | x | x | x | x | x | x | x | x |
| _cons | x | x | x | x | x | x | x | x | x | x |

**Table S36. Control variables retained in CRE Model 2 across 10 folds.** Number of days antibiotics administered.

|  | 1 | 2 | 3 | 4 | 5 | 6 | 7 | 8 | 9 | 10 |
| --- | --- | --- | --- | --- | --- | --- | --- | --- | --- | --- |
| Severe neutropenia |  |  |  |  |  |  |  |  |  |  |
| No | x | x | x |  | x | x | x | x | x | x |
| Not recorded | x |  |  | x |  |  |  |  |  |  |
| Flucloxacillin |  |  |  |  |  |  |  |  |  |  |
| No | x | x | x | x | x | x | x | x | x | x |
| Yes |  | x |  |  |  |  |  |  |  |  |
| Metronidazole_parenteral |  |  |  |  |  |  |  |  |  |  |
| No | x | x | x | x | x | x | x | x | x | x |
| Steroid_use |  |  |  |  |  |  |  |  |  |  |
| No |  |  |  |  |  | x |  |  |  |  |
| _cons | x | x | x | x | x | x | x | x | x | x |

**Table S37. Control variables retained in CRE Model 2 across 10 folds.** HIV.

|  | 1 | 2 | 3 | 4 | 5 | 6 | 7 | 8 | 9 | 10 |
| --- | --- | --- | --- | --- | --- | --- | --- | --- | --- | --- |
| Severe neutropenia |  |  |  |  |  |  |  |  |  |  |
| No | x | x | x |  |  | x |  | x | x | x |
| TB |  |  |  |  |  |  |  |  |  |  |
| No | x | x | x | x | x | x | x | x | x | x |
| No Chronic Disease |  |  |  |  |  |  |  |  |  |  |
| No | x | x | x | x | x | x | x | x | x | x |
| _cons | x | x | x | x | x | x | x | x | x | x |

**Table S38. Control variables retained in CRE Model 2 across 10 folds.** Urinary Catheter.

|  | 1 | 2 | 3 | 4 | 5 | 6 | 7 | 8 | 9 | 10 |
| --- | --- | --- | --- | --- | --- | --- | --- | --- | --- | --- |
| ippartagegp3 | x |  | x | x | x | x | x | x | x | x |
| Severe neutropenia |  |  |  |  |  |  |  |  |  |  |
| No |  |  | x | x |  |  |  |  |  | x |
| Metronidazole_parenteral |  |  |  |  |  |  |  |  |  |  |
| No |  |  | x | x |  |  |  |  |  | x |
| _cons | x | x | x | x | x | x | x | x | x | x |

**Table S39. Control variables retained in CRE Model 2 across 10 folds.** Intubated.

|  | 1 | 2 | 3 | 4 | 5 | 6 | 7 | 8 | 9 | 10 |
| --- | --- | --- | --- | --- | --- | --- | --- | --- | --- | --- |
| Ceftazidime |  |  |  |  |  |  |  |  |  |  |
| No | x | x | x | x |  | x | x | x | x | x |
| _cons | x | x | x | x | x | x | x | x | x | x |

**Table S40. Control variables retained in CRE Model 2 across 10 folds**. Hospital stay for ≥ 3 days.

|  | 1 | 2 | 3 | 4 | 5 | 6 | 7 | 8 | 9 | 10 |
| --- | --- | --- | --- | --- | --- | --- | --- | --- | --- | --- |
| Erythromycin |  |  |  |  |  |  |  |  |  |  |
| No | x |  |  | x |  | x | x |  | x |  |
| Yes |  |  |  |  | x |  |  |  |  |  |
| _cons | x | x | x | x | x | x | x | x | x | x |

**Table S41. Control variables retained in CRE Model 2 across 10 folds.** Transferred from another facility.

|  | 1 | 2 | 3 | 4 | 5 | 6 | 7 | 8 | 9 | 10 |
| --- | --- | --- | --- | --- | --- | --- | --- | --- | --- | --- |
| Kidney_disease |  |  |  |  |  |  |  |  |  |  |
| No | x | x | x |  | x |  | x | x | x |  |
| Yes |  |  |  |  |  |  |  |  |  | x |
| Severe neutropenia |  |  |  |  |  |  |  |  |  |  |
| No |  |  |  |  | x |  |  |  |  |  |
| Erythromycin |  |  |  |  |  |  |  |  |  |  |
| No |  |  |  |  | x |  |  |  |  | x |
| _cons | x | x | x | x | x | x | x | x | x | x |

**Table S42. Control variables retained in CRE Model 3 across 10 folds.** CRE outcome variable.

|  | 1 | 2 | 3 | 4 | 5 | 6 | 7 | 8 | 9 | 10 |
| --- | --- | --- | --- | --- | --- | --- | --- | --- | --- | --- |
| _cons | x | x | x | x | x | x | x | x | x | x |

**Table S43. Control variables retained in CRE Model 3 across 10 folds.** Ceftriaxone use.

|  | 1 | 2 | 3 | 4 | 5 | 6 | 7 | 8 | 9 | 10 |
| --- | --- | --- | --- | --- | --- | --- | --- | --- | --- | --- |
| Benzylpenicillin |  |  |  | x |  |  |  |  |  |  |
| No |  |  |  |  |  |  |  |  |  |  |
| Kidney_disease |  |  |  |  |  |  |  |  |  |  |
| No |  |  |  |  |  | x |  |  |  |  |
| _cons | x | x | x | x | x | x | x | x | x | x |

**Table S44. Control variables retained in CRE Model 3 across 10 folds.** Number of days antibiotics administered.

|  | 1 | 2 | 3 | 4 | 5 | 6 | 7 | 8 | 9 | 10 |
| --- | --- | --- | --- | --- | --- | --- | --- | --- | --- | --- |
| Severe neutropenia |  |  |  |  |  |  |  |  |  |  |
| No | x | x | x |  | x | x | x | x | x |  |
| Not recorded |  |  | x | x | x |  |  |  |  | x |
| Steroid_use |  |  |  |  |  |  |  |  |  |  |
| No | x | x |  | x | x | x | x |  | x |  |
| Flucloxacillin |  |  |  |  |  |  |  |  |  |  |
| No | x | x | x | x | x | x | x | x | x | x |
| Metronidazole_parenteral |  |  |  |  |  |  |  |  |  |  |
| No | x | x | x | x | x | x | x | x | x | x |
| _cons | x | x | x | x | x | x | x | x | x | x |

**Table S45. Control variables retained in CRE Model 3 across 10 folds.** HIV.

|  | 1 | 2 | 3 | 4 | 5 | 6 | 7 | 8 | 9 | 10 |
| --- | --- | --- | --- | --- | --- | --- | --- | --- | --- | --- |
| Severe neutropenia |  |  |  |  |  |  |  |  |  |  |
| No | x | x | x |  |  | x |  | x | x | x |
| TB |  |  |  |  |  |  |  |  |  |  |
| No | x | x | x | x | x | x | x | x | x | x |
| No Chronic Disease |  |  |  |  |  |  |  |  |  |  |
| No | x | x | x | x | x | x | x | x | x | x |
| _cons | x | x | x | x | x | x | x | x | x | x |

**Table S46. Control variables retained in CRE Model 3 across 10 folds.** Urinary Catheter.

|  | 1 | 2 | 3 | 4 | 5 | 6 | 7 | 8 | 9 | 10 |
| --- | --- | --- | --- | --- | --- | --- | --- | --- | --- | --- |
| ippartagegp3 | x |  | x | x | x |  | x |  |  |  |
| Severe neutropenia |  |  |  |  |  |  |  |  |  |  |
| No |  |  |  | x |  |  |  |  |  |  |
| Metronidazole_parenteral |  |  |  |  |  |  |  |  |  |  |
| No |  |  |  | x |  |  |  |  |  |  |
| _cons | x | x | x | x | x | x | x | x | x | x |

**Table S47. Control variables retained in CRE Model 3 across 10 folds**. Intubated.

|  | 1 | 2 | 3 | 4 | 5 | 6 | 7 | 8 | 9 | 10 |
| --- | --- | --- | --- | --- | --- | --- | --- | --- | --- | --- |
| Ceftazidime |  |  |  |  |  |  |  |  |  |  |
| No | x | x | x | x |  | x | x | x | x | x |
| _cons | x | x | x | x | x | x | x | x | x | x |

**Table S48. Control variables retained in CRE Model 3 across 10 folds.** Hospital stay for ≥3 days.

|  | 1 | 2 | 3 | 4 | 5 | 6 | 7 | 8 | 9 | 10 |
| --- | --- | --- | --- | --- | --- | --- | --- | --- | --- | --- |
| _cons | x | x | x | x | x | x | x | x | x | x |

**Table S49. Control variables retained in CRE Model 3 across 10 folds.** Transferred from another facility.

|  | 1 | 2 | 3 | 4 | 5 | 6 | 7 | 8 | 9 | 10 |
| --- | --- | --- | --- | --- | --- | --- | --- | --- | --- | --- |
| Kidney_disease |  |  |  |  |  |  |  |  |  |  |
| No | x | x | x | x | x |  | x | x | x | x |
| Severe neutropenia |  |  |  |  |  |  |  |  |  |  |
| No |  |  |  |  | x |  |  |  |  |  |
| Erythromycin |  |  |  |  |  |  |  |  |  |  |
| Yes |  |  |  |  | x |  |  |  |  |  |
| No |  |  |  |  |  |  |  |  |  | x |
| _cons | x | x | x | x | x | x | x | x | x | x |
